# Supplementary material for: Vascular access devices and associated complications in paediatric critical care: A prospective cohort study
Source: PLoS One. 2024 Oct 24;19(10):e0309650. doi: 10.1371/journal.pone.0309650 (PMC11500871; doi:10.1371/journal.pone.0309650)
Supplement: S1 File — (DOCX) [file pone.0309650.s001.docx]

## **Vascular Access Devices And Complications In Pediatric Critical Care: A Prospective Cohort Study**

Melany Gaetani^1-6^,
Sarah Kleiboer^7^,
Randolph Kissoon^8^,
Kristen Middaugh^1,4,6^,
Christopher S Parshuram^1-6^

^1^Child Health Evaluative Sciences, The Research Institute Hospital for Sick Children, Toronto, Ontario, Canada

^2^Departments of Health Policy, Management, and Evaluation, University of Toronto,

^3^Interdepartmental Division of Critical Care Medicine, Faculty of Medicine, University of Toronto, Toronto, Ontario, Canada

^4^Department of Critical Care Medicine, The Hospital for Sick Children, Toronto, Ontario, Canada

^5^Department of Paediatrics, The Hospital for Sick Children, Toronto, Ontario, Canada

^6^Center for Safety Research, Toronto, Ontario, Canada

7Newmarket, Ontario, Canada

8Toronto, Ontario, Canada

**Corresponding Author:**

Dr. Christopher Parshuram

Staff Physician

Department of Critical Care Medicine

Hospital for Sick Children

555 University Avenue

Toronto, Ontario, Canada

M5G1X8

[chris@sickkids.ca](mailto:chris@sickkids.ca)

**S1 Table: Outcome definitions**

| **Outcome** | **Definition** |
| --- | --- |
| **Device Complication** | One or more of device malposition, local thrombosis, infection, leakage.  extravasation, embolism, migration, vascular erosion. |
| Device malposition | Will include one or more of  [a] migration to unintended intravascular location (eg. Intra-hepatic umbilical line, peripherally inserted central catheter going into head and neck vessels rather than towards the heart). This will be apparent on radiologic examination.  [b] extravasation of infused medication or fluids into tissues, or body cavity.  [c] erosion of the device through a major vessel wall. |
| Local thrombosis | Clinical evidence of vascular obstruction or ultrasound confirmed thrombosis of the vessel where the vascular access device is within the vessel or immediately adjacent to the device, with or without related embolism. Embolism will includes distal end-organ (eg. fingers, toes) cutaneous and other infarctions. |
| Device leakage | Any description of leaking of infused fluids from the vascular access device. |
| Device infection | Documented infection at the device insertion point or of the device itself in the bloodstream (also known as catheter-associated blood stream infection) |
| **Lumen Dysfunction** | One or more of difficulty or inability to either infuse into, or aspirate blood from, the lumen. |
| Ease of infusion | Describes the nature (ease/ difficulty) of infusion into the lumen. Options are:  [1] Unobstructed: where the lumen is known to accept pushed or infused medications without difficulty. This includes situation where pumps are working well, and medications are being delivered effectively.  [2] Partial obstruction: where infusion is possible, but requires more physical force than usual or longer time than expected.  [3] Complete obstruction: where the lumen does not flush or accept infusions.  [4] Unknown: where the lumen is not being used and has not been tested. |
| Ability to aspirate | This is the ability for blood to be aspirated from the lumen. Aspiration of blood does not have to be for sampling for biochemical or other testing, but needs to be have been done to confirm this aspect of lumen function. The ability to aspirate blood from the lumen is reported by the primary nurse. Thus options are:  [1] Yes: able to aspirate blood from the lumen.  [2] No (blocked): unable to aspirate blood from the lumen.  [3] Unknown: where the ability to aspirate blood has not been recently tested, or the results of testing are unknown by the primary nurse. |

**Legend**: Intravascular devices are comprised of intra-arterial and intravenous catheters used to treat critically ill children. Devices are used to provide intravenous fluids – as maintenance or bolus fluids; transfusion of blood components; medication administration – as either intermittent or bolus doses and for measurement of intravascular pressures – arterial or venous. Device complications relate to device position, vessel thrombosis, insertion-site leakage, and catheter-related infection. Lumen malfunctions reflect compromised operations of individual lumens of devices. Device complications and lumen malfunctions may result in decisions to remove or replace devices.

**S2 Table: R packages used**

library(usethis)

library(tidyverse)

library(devtools)

library(ggpubr)

library(moments)

library(arsenal)

library(helda)

library(summarytools)

library(survival)

library(survminer)

library(scales)

library(reshape2)

library(zoo)

library(msm)

library(lubridate)

**S3 Table: Central venous catheters by line type and observed complications**

|  | **Percutaneous CVC (N = 175)** | **Umbilical Venous Catheter (n= 17)** | **Other (n = 14)** |
| --- | --- | --- | --- |
| **Total observed days, n** | 577 | 41 | 27 |
| **Complication days total, n (%)** | 82 (14) | 6 (15) | 5 (18) |
| **Complication days by type of complication** |  |  |  |
| Infection, n (%) | 3 (0.5) | - | - |
| Leakage, n (%) | 31 (5) | 2 (5) | 3 (11) |
| Malposition, n (%) | 15 (3) | 4 (10) | - |
| Thrombosis, n (%) | 33 (6) | - | 2 (7) |
| Removed during study period, n (%) | 42 (24) | 5 (29) | 3 (21) |
| Device age before removal, days, median(IQR) | 9 (5 - 17) | 2.5 (1.7 - 3.5) | 27 (20 - 32) |
| Patient age, months, median(IQR) | 3 (0.25 - 8) | **all patients <7 days* | 12.5 (0.25 - 45) |

There was a total of 206 CVCs included in this study. Other refers to tunneled central venous catheters and hemodialysis catheters.

**S4 Table: Multistate model estimated probability of devices transitioning from one state to another state over a given period of time.**

| *Device = CVC* |  |  | *To* |  |
| --- | --- | --- | --- | --- |
| *P (14 days)* |  | **No Complication** | **Complication** | **Removal** |
|  | **No Complication** | 0.4929948 | 0.06316831 | 0.4438369 |
| *From* | **Complication** | 0.248595 | 0.03422481 | 0.7171802 |
|  | **Removal** | 0 | 0 | 1 |
| *Device = PICC* |  |  | *To* |  |
| *P (30 days)* |  | **No Complication** | **Complication** | **Removal** |
|  | **No Complication** | 0.5874584 | 0.1524347 | 0.2601069 |
| *From* | **Complication** | 0.4676907 | 0.1303878 | 0.4019215 |
|  | **Removal** | 0 | 0 | 1 |
| *Device = ART* |  |  | *To* |  |
| *P (7 days)* |  | **No Complication** | **Complication** | **Removal** |
|  | **No Complication** | 0.6326084 | 0.05752107 | 0.3098705 |
| *From* | **Complication** | 0.2289855 | 0.15190242 | 0.619112 |
|  | **Removal** | 0 | 0 | 1 |

S4 Table estimates the probabilities of devices transitioning from one state to another over a given period of time. The probability of line removal within 14 days of a complication is 71% of CVCs. Within 30 days, the probability of removing a PICC following complications is 40% and in arterial lines with a complication, the probability of removing the device at 7 days is 62%. ART = Arterial Line, CVC = central venous catheter; PICC = Peripherally inserted central catheter

**S5 Table: Multistate model covariates effects by device and transition states**

| *Device = CVC* |  |  |  |  |  |
| --- | --- | --- | --- | --- | --- |
| **Transition 1 → 2 (No Complication to Complication)** | | |  |  |  |
| Covariate | Relative Rate | 95% CI | | | p-value |
| Weight | 0.91 | 0.80 | - | 1.03 | 0.149 |
| Thromboembolic prophylactic treatment | 0.92 | 0.20 | - | 4.27 | 0.919 |
| Thromboembolic treatment | 0.67 | 0.30 | - | 1.49 | 0.326 |
| **Transition 1 → 3 (No Complication to Removal)** | |  |  |  |  |
| Covariate | Relative Rate | 95% CI | | | p-value |
| Weight | 0.91 | 0.80 | - | 1.03 | 0.15 |
| Thromboembolic prophylactic treatment | 0.92 | 0.20 | - | 4.27 | 0.92 |
| Thromboembolic treatment | 0.67 | 0.30 | - | 1.49 | 0.33 |
| **Transition 2 → 1 (Complication to No Complication)** | | |  |  |  |
| Covariate | Relative Rate | 95% CI | | | p-value |
| Weight | 0.89 | 0.72 | - | 1.08 | 0.24 |
| Thromboembolic prophylactic treatment | 1.79 | 0.33 | - | 9.69 | 0.50 |
| Thromboembolic treatment | 1.20 | 0.41 | - | 3.51 | 0.74 |
| **Transition 2 → 3 (Complication to Removal)** | |  |  |  |  |
| Covariate | Relative Rate | 95% CI | | | p-value |
| Weight | 0.79 | 0.62 | - | 0.9996 | 0.05 |
| Thromboembolic prophylactic treatment |  |  |  |  |  |
| Thromboembolic treatment | 0.64 | 0.26 | - | 1.54 | 0.32 |

| *Device = PICC* |  |  |  |  |  |
| --- | --- | --- | --- | --- | --- |
| **Transition 1 → 2 (No Complication to Complication)** | | |  |  |  |
| Covariate | Relative Rate | 95% CI | | | p-value |
| Weight | 0.99 | 0.88 | - | 1.13 | 0.98 |
| Thromboembolic prophylactic treatment |  |  |  |  |  |
| Thromboembolic treatment | 0.79 | 0.43 | - | 1.45 | 0.45 |
| **Transition 1 → 3 (No Complication to Removal)** | |  |  |  |  |
| Covariate | Relative Rate | 95% CI | | | p-value |
| Weight | 1.09 | 0.90 | - | 1.32 | 0.38 |
| Thromboembolic prophylactic treatment |  |  |  |  |  |
| Thromboembolic treatment | 0.54 | 0.14 | - | 2.07 | 0.37 |
| **Transition 2 → 1 (Complication to No Complication)** | | |  |  |  |
| Covariate | Relative Rate | 95% CI | | | p-value |
| Weight | 0.92 | 0.74 | - | 1.13 | 0.43 |
| Thromboembolic prophylactic treatment |  |  |  |  |  |
| Thromboembolic treatment | 0.65 | 0.29 | - | 1.45 | 0.29 |
| **Transition 2 → 3 (Complication to Removal)** | |  |  |  |  |
| Covariate | Relative Rate | 95% CI | | | p-value |
| Weight | 1.05 | 0.89 | - | 1.24 | 0.55 |
| Thromboembolic prophylactic treatment |  |  |  |  |  |
| Thromboembolic treatment | 0.16 | 0.04 | - | 0.61 | 0.01 |

| *Device = ART* |  |  |  |  |  |
| --- | --- | --- | --- | --- | --- |
| **Transition 1 → 2 (No Complication to Complication)** | | |  |  |  |
| Covariate | Relative Rate | 95% CI | | | p-value |
| Weight | 0.81 | 0.60 | - | 1.10 | 0.18 |
| Thromboembolic prophylactic treatment | 0.52 | 0.06 | - | 4.30 | 0.54 |
| Thromboembolic treatment | 0.44 | 0.16 | - | 1.17 | 0.10 |
| **Transition 1 → 3 (No Complication to Removal)** | |  |  |  |  |
| Covariate | Relative Rate | 95% CI | | | p-value |
| Weight | 0.99 | 0.89 | - | 1.10 | 0.86 |
| Thromboembolic prophylactic treatment | 0.46 | 0.06 | - | 3.60 | 0.46 |
| Thromboembolic treatment | 0.64 | 0.30 | - | 1.38 | 0.26 |
| **Transition 2 → 1 (Complication to No Complication)** | | |  |  |  |
| Covariate | Relative Rate | 95% CI | | | p-value |
| Weight | 0.67 | 0.27 | - | 1.70 | 0.40 |
| Thromboembolic prophylactic treatment |  |  |  |  |  |
| Thromboembolic treatment | 0.24 | 0.04 | - | 1.47 | 0.12 |
| **Transition 2 → 3 (Complication to Removal)** | |  |  |  |  |
| Covariate | Relative Rate | 95% CI | | | p-value |
| Weight | 0.70 | 0.38 | - | 1.32 | 0.28 |
| Thromboembolic prophylactic treatment | 1.54 | 0.13 | - | 18.57 | 0.73 |
| Thromboembolic treatment | 1.48 | 0.29 | - | 7.63 | 0.64 |

**S1 Figure: Multistate model transitions**

Device removal

Device with complication

Device without complication

**S2 Figure: Time to first lumen complication by volume of infusate**

**
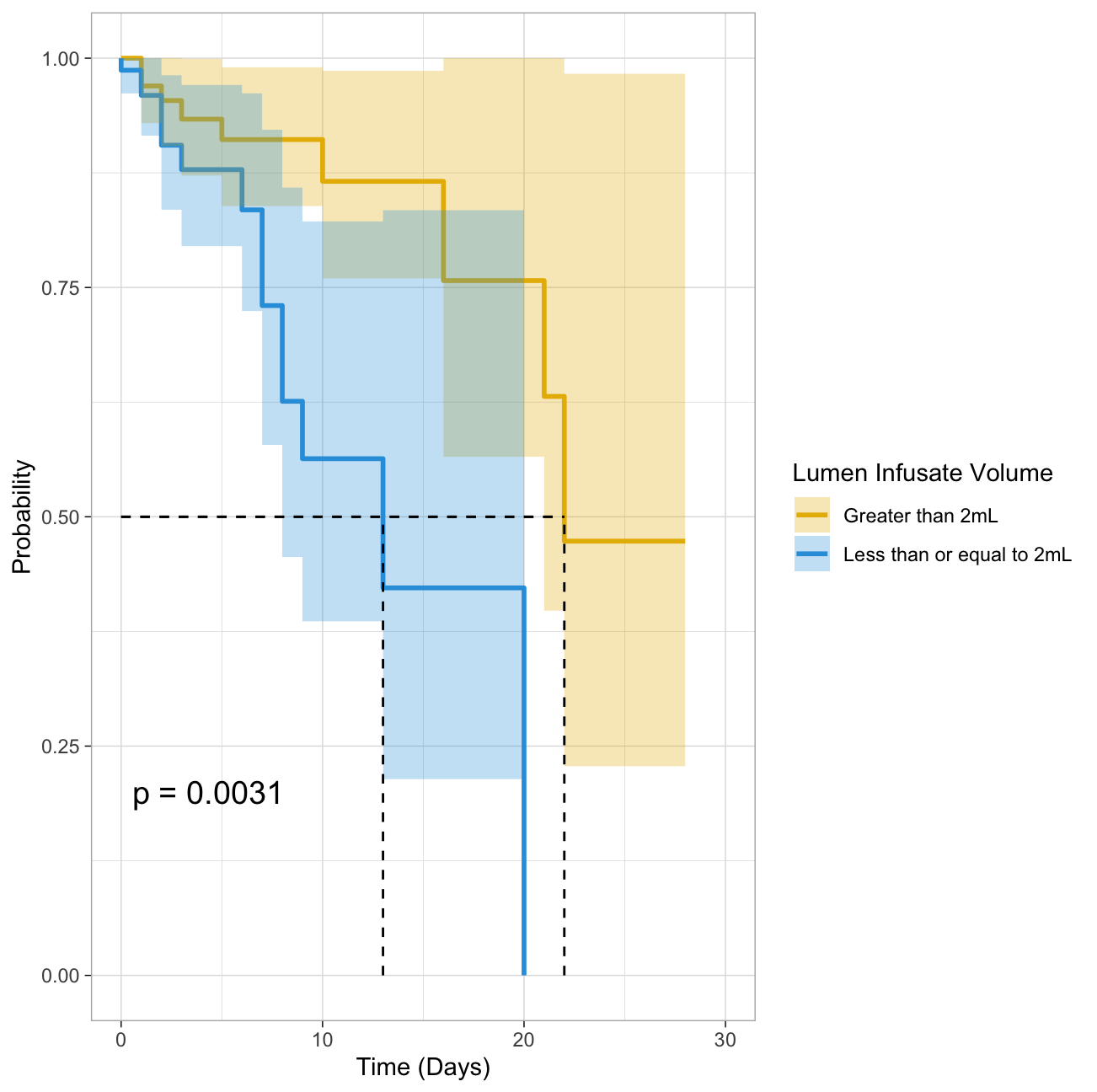
**

S1 figure shows the time to first lumen complication in CVCs by volume of infusate into each lumen at the time observation. Time to first complication was statically significantly different (p-value <0.05) between lumens running 2mL or less and those with larger volumes of fluid.

STROBE Statement—Checklist of items that should be included in reports of ***cohort studies***

**Item**

**No Recommendation**

**Title and abstract** 1

**Introduction**

Background/rationale

Objectives

2

3

Explain the scientific background and rationale for the investigation being reported

State specific objectives, including any prespecified hypotheses

1. Indicate the study’s design with a commonly used term in the title or the abstract
2. Provide in the abstract an informative and balanced summary of what was done and what was found

**Methods**

Study design 4 Present key elements of study design early in the paper

Setting 5 Describe the setting, locations, and relevant dates, including periods of recruitment, exposure, follow-up, and data collection

Participants 6

1. Give the eligibility criteria, and the sources and methods of selection of participants. Describe methods of follow-up
2. For matched studies, give matching criteria and number of exposed and unexposed

Variables 7 Clearly define all outcomes, exposures, predictors, potential confounders, and effect modifiers. Give diagnostic criteria, if applicable

Data sources/ measurement

8* For each variable of interest, give sources of data and details of methods of assessment (measurement). Describe comparability of assessment methods if there is more than one group

Bias 9 Describe any efforts to address potential sources of bias

Study size 10 Explain how the study size was arrived at

Quantitative variables 11 Explain how quantitative variables were handled in the analyses. If applicable,

describe which groupings were chosen and why

Statistical methods 12

**Results**

Participants 13*

Descriptive data 14*

1. Describe all statistical methods, including those used to control for confounding
2. Describe any methods used to examine subgroups and interactions
3. Explain how missing data were addressed
4. If applicable, explain how loss to follow-up was addressed
5. Describe any sensitivity analyses
6. Report numbers of individuals at each stage of study—eg numbers potentially eligible, examined for eligibility, confirmed eligible, included in the study, completing follow-up, and analysed
7. Give reasons for non-participation at each stage
8. Consider use of a flow diagram
9. Give characteristics of study participants (eg demographic, clinical, social) and information on exposures and potential confounders
10. Indicate number of participants with missing data for each variable of interest
11. Summarise follow-up time (eg, average and total amount)

Outcome data 15* Report numbers of outcome events or summary measures over time

Main results 16

1. Give unadjusted estimates and, if applicable, confounder-adjusted estimates and their precision (eg, 95% confidence interval). Make clear which confounders were adjusted for and why they were included
2. Report category boundaries when continuous variables were categorized
3. If relevant, consider translating estimates of relative risk into absolute risk for a meaningful time period

Other analyses 17 Report other analyses done—eg analyses of subgroups and interactions, and

sensitivity analyses

**Discussion**

Key results 18 Summarise key results with reference to study objectives

Limitations 19 Discuss limitations of the study, taking into account sources of potential bias or

imprecision. Discuss both direction and magnitude of any potential bias Interpretation 20 Give a cautious overall interpretation of results considering objectives, limitations,

multiplicity of analyses, results from similar studies, and other relevant evidence Generalisability 21 Discuss the generalisability (external validity) of the study results

**Other information**

Funding 22 Give the source of funding and the role of the funders for the present study and, if

applicable, for the original study on which the present article is based

*Give information separately for exposed and unexposed groups.

**Note:** An Explanation and Elaboration article discusses each checklist item and gives methodological background and published examples of transparent reporting. The STROBE checklist is best used in conjunction with this article (freely available on the Web sites of PLoS Medicine at [http://www.plosmedicine.org/,](http://www.plosmedicine.org/) Annals of Internal Medicine at [http://www.annals.org/,](http://www.annals.org/) and Epidemiology at [http://www.epidem.com/).](http://www.epidem.com/)) Information on the STROBE Initiative is available at [http://www.strobe-statement.org.](http://www.strobe-statement.org/)
