# Supplementary material for: Vascular access devices and associated complications in paediatric critical care: A prospective cohort study
Source: PLoS One. 2024 Oct 24;19(10):e0309650. doi: 10.1371/journal.pone.0309650 (PMC11500871; doi:10.1371/journal.pone.0309650)
Supplement: Attachment — Submitted filename: S_therapies_PLOS_R2R cp.docx [file pone.0309650.s002.docx]

**Response to Reviewers**

**The authors wish to thank the reviewers for their thoughtful comments and suggestions, and to the editors for the providing us the opportunity to respond. We believe that the manuscript has improved as a consequence. Please find our edits and additions to each response outlined below.**

**Thank you.**

**Sincerely,**

**Melany Gaetani, Sarah Kleiboer, Randolph Kissoon, Kristen Middaugh, Christopher S Parshuram.**

**Journal Requirements:**

1. When submitting your revision, we need you to address these additional requirements.

Please ensure that your manuscript meets PLOS ONE's style requirements, including those for file naming. The PLOS ONE style templates can be found at 
<https://journals.plos.org/plosone/s/file?id=wjVg/PLOSOne_formatting_sample_main_body.pdf> and 
<https://journals.plos.org/plosone/s/file?id=ba62/PLOSOne_formatting_sample_title_authors_affiliations.pdf>

2. Please include captions for your Supporting Information files at the end of your manuscript, and update any in-text citations to match accordingly. Please see our Supporting Information guidelines for more information: <http://journals.plos.org/plosone/s/supporting-information>.

Thank you. These have been updated.

**Reviewers' comments:**

***Reviewer #1:***

Manuscript was presented as stated in the abstract. The graphs were easy to interpret and consistent with the objectives in the manuscript. The conclusion brought in the thought that as clinicians and researchers need to focus future investigation on the cause, effect and interventions necessary to promote best practice.

Thank you.

***Reviewer #2:***

Parshuram and colleagues provide a study investigating the occurrence of problems associated with the utilization of venous access devices in children admitted to the intensive care units at the Sick Children Hospital in Toronto.
This study includes a significant number of patients and the duration of their enrollment is sufficiently long.

However, it contains multiple deformations, mistakes, and conceptual flaws that negatively impact its quality in terms of methodology and structure/conceptual framework.

[1] To begin with, discussing the "complications" of vascular access in such a general manner is completely inappropriate and inaccurate. Comparing peripheral venous access without providing appropriate categorization is not feasible.

Thank you. We agree with the reviewer that combining venous and arterial access *data* into a single group is problematic. We have been careful to separate out arterial and venous access devices in all analyses, subcategorized venous access into central and peripheral venous access types and *further* subcategorized central venous access into directly or peripherally inserted central (PICC) venous devices.

In response to the reviewers comments we:

**[i] Revised the methods** to better represent these separations and improve clarity for readers.

**[ii] A new Table S3 has been added** to more distinctly report the sub-types of central venous access devices observed. Given the modest numbers of tunneled and umbilical catheters we have resisted the temptation to conduct separate analyses of these lines.

**[iii] Conducted a sensitivity analysis.** We re-ran the main study analysis including only CVL known to be percutaneously inserted temporary CVL (thus excluding peripherally inserted central catheters, umbilical and tunneled CVL). The probability of removal after a complication changed by <3%. This has been documented with the results of the main analysis and is supported by the observed frequencies of complications in table S3.

Thank you.

[2] I would want to emphasize to the authors that peripheral venous access encompasses several methods such as a butterfly needle, a venous cannula, and a midline. This is in contrast to central and arterial access methods, and even includes intraosseous access.

We agree with the reviewer that peripheral intravenous access represents an important subset of intravascular devices and acknowledge that readers will have experiences using different forms of peripheral intravenous access.

On re-review of the data our anecdotal experience that butterfly needles and interosseus devices were not amongst the types of vascular access in this study has been reinforced. First, the gauge sizes recorded for the PIVs do not include available butterfly needle gauges (21, 23, 25G). Butterfly needles are used for venepuncture and are almost never used for longer term use in our anecdotal observation.

In response to these comments we have:

**[i] provided more local context** to better inform interpretations: highlighted observations drawn from the data and shared our local knowledge to reassure readers that peripheral venous access is unlikely to be of butterfly needle or midline type.

**[ii] revised the limitations** to acknowledge that these devices were not part of the current study and that the generalizability of current results should not be assumed.

[3] This collection encompasses even arterial cannulae.

Thank you. All data analysis and figures pertaining specifically to devices and lumens differentiate arterial and venous vascular access.

[4] The complications associated with central venous access are distinct from those of peripheral venous access.

Thank you. We agree with the reviewer that peripheral intravenous access represents an important subset of intravascular devices and were analyzed separately from other devices.

**[i]** **clarified the methods :** to recognize the uniqueness of peripheral venous access – such that removal was considered a complication – given the relatively low rates of ‘voluntary removal’ of this type of device.

**[ii] added** acknowledgment of the uniqueness of peripheral IV access by adding a citation of a systematic review of complications in adult patients ^1^.

***Reference:***

1. *Marsh N, Webster J, Ullman AJ, Mihala G, Cooke M, Chopra V, Rickard CM. Peripheral intravenous catheter non-infectious complications in adults: A systematic review and meta-analysis. J Adv Nurs. 2020 Dec;76(12):3346-3362. doi: 10.1111/jan.14565. Epub 2020 Oct 5. PMID: 33016412.)*

Moreover, even within the realm of central venous access, the specific type of access being referred to is not well defined. It is important to properly categorize and differentiate between umbilical catheters, non-tunneled cuffed/non-cuffed central venous catheters (CICCs), tunneled cuffed/non-cuffed CICCs, peripherally inserted central catheters (PICCs), and ports. Failing to do so would result in a disorganized collection of these devices. Furthermore, it is worth noting that dialysis and pheresis catheters are entirely omitted.

We agree with the reviewer that these types of lines are important to be differentiated. In response we clarified the methods, added a table with the sub-types of venous access, added to results, conducted a sensitivity analysis, and acknowledged the modest numbers of sub-categories of these devices in the limitations. Please see our responses to point [1] above.

Moreover, there is no categorization of patients based on their age. For instance, in newborns, umbilical catheters are utilized. However, if these catheters remain in place for over 7 days or are poorly positioned, they can increase the likelihood of portal vein thrombosis. Additionally, epicutaneous-caval catheters are also employed. these examples are to say that depending on age and pathology the complications with vascular access can be different.

Thank you. The reviewers point about the age of lines inserted into umbilical vessels highlights several important considerations related to these lines.

There were several reasons we chose to focus on patient weight rather than age in the multistate model. First, these two variables are closely related to one another – and especially in younger patients – such as this cohort of patients who are largely under 14 months old. Second, from first principles and some literature, weight is good and sometimes better predictor of vessels diameter, lengths and depth (second only to height which we did not collect)^1,2^.

In response we have

**[i] clarified scientific rationale:** included the two references to better provide scientific justification for the use of weight in predictive modelling.

**[ii] included a description of age** vs line type in the results (Table S3) and shown the age of unbilical lines was less than the 7 days described by the reviewer as risk-laden.

**[iii] added to the results** the details of the regression analysis of risk factors for thrombosis including that we found age and weight were collinear. We have added all covariates included in the multistate model for clarity in the results.

**[iv] increased details about the types of vascular access devices:** We have also listed all types of catheters include in this study in the results and a table within the supplementary data in hopes to add greater transparency and precision into the interpretation and external validity of these results. This provides readers with greater understanding about the catheters that were and were not used – including that epicutaneous-caval catheters were not used.

**[v] Limitations:** We acknowledge the range of devices used in this single center as a potential limitation to generalizability the lack of data pertaining to the underlying pathology of the individual patients. We agree more detailed description of patient pathologies will be a valuable component of future work.

***References:***

1. *López Álvarez, J. M., Pérez Quevedo, O., Santana Cabrera, L., Escot, C. R., Loro Ferrer, J. F., Lorenzo, T. R., & Limiñana Cañal, J. M. (2017). Vascular ultrasound in pediatrics: estimation of depth and diameter of jugular and femoral vessels. Journal of Ultrasound, 20, 285-292.*
2. *Fang, Xue-qi Master; Zhang, Hao Bachelor; Zhou, Ji-ming Master∗. Angiography in pediatric patients: Measurement and estimation of femoral vessel diameter. Medicine 99(31):p e21486, July 31, 2020. | DOI: 10.1097/MD.0000000000021486*

The authors thank the reviewers for taking the time to review, for the thoughtful comments and suggestions, and thank the editor for allowing us the opportunity to respond. We believe the work has been strengthened as a consequence. Thank you

C Parshuram and M Gaetani

For the authors.
