# Supplementary material for: Vascular access devices and associated complications in paediatric critical care: A prospective cohort study
Source: PLoS One. 2024 Oct 24;19(10):e0309650. doi: 10.1371/journal.pone.0309650 (PMC11500871; doi:10.1371/journal.pone.0309650)
Supplement: Attachment — Submitted filename: Therapies_RSR_2cp.docx [file pone.0309650.s003.docx]

**Response to Reviewers**

*The authors wish to thank the reviewers for their thoughtful comments and suggestions, and to the editors for the providing us the opportunity to respond. We believe that the manuscript has improved as a consequence.*

*Please find our edits and additions to each addressable point in the response outlined below.*

Thank you.

Sincerely,

Melany Gaetani, Sarah Kleiboer, Randolph Kissoon, Kristen Middaugh, Christopher S Parshuram.

PONE-D-23-38349R1

Vascular Access Devices and Associated Complications in Paediatric Critical Care: A Prospective Cohort Study

PLOS ONE

Reviewers' comments:

Reviewer's Responses to Questions

**Comments to the Author**

1. If the authors have adequately addressed your comments raised in a previous round of review and you feel that this manuscript is now acceptable for publication, you may indicate that here to bypass the “Comments to the Author” section, enter your conflict of interest statement in the “Confidential to Editor” section, and submit your "Accept" recommendation.

Reviewer #2: (No Response)

Reviewer #3: All comments have been addressed

*Thank you.*

2. Is the manuscript technically sound, and do the data support the conclusions?

The manuscript must describe a technically sound piece of scientific research with data that supports the conclusions. Experiments must have been conducted rigorously, with appropriate controls, replication, and sample sizes. The conclusions must be drawn appropriately based on the data presented.

Reviewer #2: No

Reviewer #3: Partly

*Thank you.*

3. Has the statistical analysis been performed appropriately and rigorously?

Reviewer #2: No

Reviewer #3: Yes

*Thank you. Please see responses to specific comments below.*

4. Have the authors made all data underlying the findings in their manuscript fully available?

The [PLOS Data policy](https://can01.safelinks.protection.outlook.com/?url=http%3A%2F%2Fwww.plosone.org%2Fstatic%2Fpolicies.action%23sharing&data=05%7C02%7Cmelany.gaetani%40sickkids.ca%7C01f994355811413c09df08dc703f7fee%7C3961553ff47e49eb9f6ccf8518914e9a%7C0%7C0%7C638508665233425698%7CUnknown%7CTWFpbGZsb3d8eyJWIjoiMC4wLjAwMDAiLCJQIjoiV2luMzIiLCJBTiI6Ik1haWwiLCJXVCI6Mn0%3D%7C0%7C%7C%7C&sdata=F8B7%2BuAZL925gQyKpMLRw%2BakIM7w%2F9yI1%2FVnOYDEuEs%3D&reserved=0) requires authors to make all data underlying the findings described in their manuscript fully available without restriction, with rare exception (please refer to the Data Availability Statement in the manuscript PDF file). The data should be provided as part of the manuscript or its supporting information, or deposited to a public repository. For example, in addition to summary statistics, the data points behind means, medians and variance measures should be available. If there are restrictions on publicly sharing data—e.g. participant privacy or use of data from a third party—those must be specified.

Reviewer #2: Yes

Reviewer #3: Yes

5. Is the manuscript presented in an intelligible fashion and written in standard English?

PLOS ONE does not copyedit accepted manuscripts, so the language in submitted articles must be clear, correct, and unambiguous. Any typographical or grammatical errors should be corrected at revision, so please note any specific errors here.

Reviewer #2: Yes

Reviewer #3: No

*Thank you. We believe the written English is sensible from grammatical and syntactic standpoints. We are happy to revise further in copy-editing or at earlier stage to improve clarity of the messages.*

6. Review Comments to the Author

Please use the space provided to explain your answers to the questions above. You may also include additional comments for the author, including concerns about dual publication, research ethics, or publication ethics. (Please upload your review as an attachment if it exceeds 20,000 characters)

**Reviewer #2: After thoroughly reviewing the revision suggested by the Authors following my feedback on the initial version of the work, I found that while the revisions and accompanying explanations provided by the Authors are pertinent and sufficient, I still find it challenging to identify the coherent structure of the work. The combination of various types of venous accesses, including peripheral cannulae, arterial cannulae, central venous catheters, umbilical catheters, and dialysis catheters, along with the accompanying analysis of associated issues, appears to be burdensome and disorganized from a conceptual standpoint. Moreover, it may potentially lead to confusion and errors for readers.**

*We thank the reviewer for their ongoing engagement in the review process and acknowledge that the various combinations of vascular access devices in critical care and their real-time management are complex.*

*The overarching goal of the work was (and is) to categorize and evaluate this complexity without the sacrifice of clinically relevant information that often accompanies ‘simplification’. We believe this is one feature that differentiates this work from other work in the field and reflects nuances of devices considered by clinicians in practice at the bedside.*

*In response to this over-arching suggestion we have:*

*[1] revised the nomenclature to be more consistent (following reviewer 2 suggestion)*

*[2] Revised supplementary table 1 to include a legend explaining context of use.*

*[3] revised the discussion to include acknowledgement of the complexity and the need for greater understanding – first through observational studies, then interventional work – to inform best practice with objective evidence.*

**For certain categories such as PICC and central venous catheters, the method of insertion is not specified, which is a crucial component in the development of problems.**

*Thank you. We did not abstract this – due to limited availability.*

*In response we have*

*[1] clarified these points in the methods.*

“*In this center, CVCs and PICCs are most commonly inserted using ultrasound guided Seldinger technique, however a significant proportion of patients have pre-existing temporary or long term vascular access devices on admission.”*

*[2] emphasized this as in the revised limitations section.*

*Thank you.*

**Reviewer #3: It's intriguing to note that vascular access utilization, a crucial aspect of Pediatric Critical Care, needs to be extensively explored in the literature. This could be due to various factors, including hospital liability. However, it's important to shift the focus from the complications associated with vascular access to its necessity, a perspective that your work can significantly contribute to. The manuscript is a brief study of how long the catheters last until they are removed. I suggest a significant restructuring.**

**[1] First, an appropriate introduction to what kind of vascular access we need: The authors can read the MAGIC (miniMAGIC) protocol and understand and better express why we need specific types of access. Ullman AJ, Bernstein SJ, Brown E, Aiyagari R, Doellman D, Faustino VS, et al. The Michigan appropriateness guide for intravenous catheters in pediatrics: miniMAGIC. Pediatrics. (2020) 145(Suppl 3):S269–84. Then, they can review a similar study, such as the one presented: Peripheral vascular access as exclusive access mode in the pediatric intensive care unit. (Armstrong). With a comprehensive understanding of the existing literature and the recommendations in the field, I am confident that your manuscript can be adjusted to make a significant and meaningful contribution.**

*Thank you. As clinicians with over 30 years of critical care experience in busy quaternary PICUs, we appreciate the sentiments expressed by this reviewer. Our research program includes considerations of the nature of parenteral therapies, intravascular fluids, drug-drug compatibility and this work evaluating the nature of vascular access devices, their function and the associated patient-related complications.*

*We have incorporated descriptions in the cited work in the revised manuscript. Thank you.*

*“…, this work demonstrates the complexity of vascular access in critically ill children and adds to the modest body of literature, with a level of detail not previously described. Selection of an ‘ideal’ device is complicated by the challenges of prospectively predicting the duration of critical illness, time-sensitive needs to provide parenteral therapies, the logistics of vascular access insertion, and the complexities of drug-drug compatibility. Vascular access device complications are frequently cited a rationale to minimize their use, to guide selection of device type and to reduce the need for replacement. Current recommendations are based on a limited number of studies, expert opinion and incomplete descriptions, highlighting the need for a broader evidence base to inform vascular access practice. Balancing the goal of ideal function versus the opportunity costs of replacement, non-function, re-insertion or removal will continue to require thoughtful, recursive, consideration tailored to the evolving needs of each patient and the best available clinical prediction. Our findings of continued use and complication resolution reflect additional complexity clinicians must integrate into bedside decision-making about vascular access devices in critically ill paediatric patients.”*
 **[2] The etiology of removal has to be well-defined.**

*We agree with the reviewer that the etiology of removal is an important component of vascular devices however the objectives of this study were to describe the frequency, nature and timing of vascular access device (VAD)* ***use, device complications and lumen dysfunction.*** *Removal was a secondary consideration****.***

*“Fourth, we also did not describe the reason for removal or long-term complications of access devices. The tracking of reasons for removal were not feasible with available study resources. Future work to delineate reasons for removal may help identify delayed and premature removal and associated clinical consequences.”*

**[3] What is a catheter clot, a vein clot, an infection, etc.?**

*Thank you. We agree detailed descriptions of interpretations of ‘clot’ are important and can be found in the supplementary material in* ***S1 Table: outcome definitions.*** *These include functional obstruction of a lumen, and vessel associated thrombosis – with ultrasound confirmed vascular obstruction.*

**[4] But again, once I see a completely revised version, more in line with what has been published in the field, I will be happy to assist. I need the authors’ help to recommend or give specific instructions.**

*Thank you. We leave the decision about the need for further revisions to the manuscript with the editors, and here reflect that the publication of work that is ‘in line’ with previous authors thinking may limit diversity of perspectives and inhibit advances in understanding.*  **[5] Though 60-80% of hospitalized patients worldwide will have an intravascular device placed during hospitalization. What is the purpose of this statement? The study is for the ICU patients, and I am sure the authors will agree that they don’t have 20-40% admissions without vascular access in their ICU. Please either remove it or provide the appropriate ICU vascular access rate.**

*Our initial statement was intended to provide broader context about the use of vascular access devices in hospitalized patients. We have revised the sentence.*

*“Intravascular devices are placed in the vast majority of critically patients worldwide and up to 80% of critically ill patients have central venous devices [6-8].” Thank you.* **[6] Specific to peripheral intravenous lines, removal was also considered a device complication, given the relatively low ‘voluntary removal’ of peripheral venous access in intensive care. What does this statement mean? Should patients stay in ICU when they don’t even need vascular access?**

*Thank you. ‘Removal’ was used to indicate that the specific peripheral intravenous device being followed had been removed. There are no other intended implications. Accordingly the removal of a PIV does not imply lack of other vascular access nor is it intended to be a statement about a given patients ‘need’ for ICU admission.*

**[7] central venous lines(CVL) = let’s call them what they are Central venous catheters or CVCs**

*This has been revised. Thank you.* **[8] The most common complication to occur amongst PIVs was removal. Is removal a complication? Are you thinking about dislodgement?**

*Thank you. Given our direct observations of the low rate of voluntary removal of (functional) PIV access in our paediatric ICU we have counted all removals as a device complications. In response we have:*

*[1] clarified the definition and provided rationale in the methods.*

*[2] added to the limitations.*

*We agree that better understanding the various reasons for PIV removal could be important in future work in this and other ICUs.*

7. PLOS authors have the option to publish the peer review history of their article ([what does this mean?](https://can01.safelinks.protection.outlook.com/?url=https%3A%2F%2Fjournals.plos.org%2Fplosone%2Fs%2Feditorial-and-peer-review-process%23loc-peer-review-history&data=05%7C02%7Cmelany.gaetani%40sickkids.ca%7C01f994355811413c09df08dc703f7fee%7C3961553ff47e49eb9f6ccf8518914e9a%7C0%7C0%7C638508665233432860%7CUnknown%7CTWFpbGZsb3d8eyJWIjoiMC4wLjAwMDAiLCJQIjoiV2luMzIiLCJBTiI6Ik1haWwiLCJXVCI6Mn0%3D%7C0%7C%7C%7C&sdata=pBqIdP4yXkTjiGWNY3AeSZ03%2F4pg0A%2BHpzIVYbNTU%2BM%3D&reserved=0)). If published, this will include your full peer review and any attached files.


If you choose “no”, your identity will remain anonymous but your review may still be made public.


**Do you want your identity to be public for this peer review?** For information about this choice, including consent withdrawal, please see our [Privacy Policy](https://can01.safelinks.protection.outlook.com/?url=https%3A%2F%2Fwww.plos.org%2Fprivacy-policy&data=05%7C02%7Cmelany.gaetani%40sickkids.ca%7C01f994355811413c09df08dc703f7fee%7C3961553ff47e49eb9f6ccf8518914e9a%7C0%7C0%7C638508665233441191%7CUnknown%7CTWFpbGZsb3d8eyJWIjoiMC4wLjAwMDAiLCJQIjoiV2luMzIiLCJBTiI6Ik1haWwiLCJXVCI6Mn0%3D%7C0%7C%7C%7C&sdata=UVOeGuauodJfln%2F2cwQ%2FFAUR59ba20C6Z4PvtwQjYLU%3D&reserved=0).

Reviewer #2: No

Reviewer #3: **Yes:** Thomas Spentzas

The authors thank the reviewers for their second review and editorial team for their stewardship of this process.

Thank you for your continued consideration.

C Parshuram and M Gaetani

For the authors.
