# Supplementary material for: Vascular access devices and associated complications in paediatric critical care: A prospective cohort study
Source: PLoS One. 2024 Oct 24;19(10):e0309650. doi: 10.1371/journal.pone.0309650 (PMC11500871; doi:10.1371/journal.pone.0309650)
Supplement: Attachment — Submitted filename: R5_R2R_Therapies.docx [file pone.0309650.s004.docx]

**Response to Reviewers**

**The authors wish to thank the reviewers for their thoughtful comments and suggestions, and to the editors for the providing us the opportunity to respond. We believe that the manuscript has improved as a consequence. Please find our edits and additions to each response outlined below.**

**Thank you.**

**Sincerely,**

**Melany Gaetani, Sarah Kleiboer, Randolph Kissoon, Kristen Middaugh, Christopher S Parshuram.**

PONE-D-23-38349R2

Vascular Access Devices and Associated Complications in Paediatric Critical Care: A Prospective Cohort Study

PLOS ONE

Dear Dr. Parshuram,

Thank you for submitting your manuscript to PLOS ONE. After careful consideration, we feel that it has merit but does not fully meet PLOS ONE’s publication criteria as it currently stands. Therefore, we invite you to submit a revised version of the manuscript that addresses the points raised during the review process.
Please revise.

Please submit your revised manuscript by Sep 02 2024 11:59PM. If you will need more time than this to complete your revisions, please reply to this message or contact the journal office at [plosone@plos.org](mailto:plosone@plos.org). When you're ready to submit your revision, log on to https://www.editorialmanager.com/pone/ and select the 'Submissions Needing Revision' folder to locate your manuscript file.

Please include the following items when submitting your revised manuscript:

- A rebuttal letter that responds to each point raised by the academic editor and reviewer(s). You should upload this letter as a separate file labeled 'Response to Reviewers'.
- A marked-up copy of your manuscript that highlights changes made to the original version. You should upload this as a separate file labeled 'Revised Manuscript with Track Changes'.
- An unmarked version of your revised paper without tracked changes. You should upload this as a separate file labeled 'Manuscript'.

If you would like to make changes to your financial disclosure, please include your updated statement in your cover letter. Guidelines for resubmitting your figure files are available below the reviewer comments at the end of this letter.

If applicable, we recommend that you deposit your laboratory protocols in protocols.io to enhance the reproducibility of your results. Protocols.io assigns your protocol its own identifier (DOI) so that it can be cited independently in the future. For instructions see: [https://journals.plos.org/plosone/s/submission-guidelines#loc-laboratory-protocols](https://can01.safelinks.protection.outlook.com/?url=https%3A%2F%2Fjournals.plos.org%2Fplosone%2Fs%2Fsubmission-guidelines%23loc-laboratory-protocols&data=05%7C02%7Cmelany.gaetani%40sickkids.ca%7C738234050cea4e39418308dca864c63b%7C3961553ff47e49eb9f6ccf8518914e9a%7C0%7C0%7C638570397978353899%7CUnknown%7CTWFpbGZsb3d8eyJWIjoiMC4wLjAwMDAiLCJQIjoiV2luMzIiLCJBTiI6Ik1haWwiLCJXVCI6Mn0%3D%7C0%7C%7C%7C&sdata=LAqs3eUM9YymxP4P3fu9r3NizzGGHQ19%2FttHlY%2FcfDs%3D&reserved=0). Additionally, PLOS ONE offers an option for publishing peer-reviewed Lab Protocol articles, which describe protocols hosted on protocols.io. Read more information on sharing protocols at [https://plos.org/protocols?utm_medium=editorial-email&utm_source=authorletters&utm_campaign=protocols](https://can01.safelinks.protection.outlook.com/?url=https%3A%2F%2Fplos.org%2Fprotocols%3Futm_medium%3Deditorial-email%26utm_source%3Dauthorletters%26utm_campaign%3Dprotocols&data=05%7C02%7Cmelany.gaetani%40sickkids.ca%7C738234050cea4e39418308dca864c63b%7C3961553ff47e49eb9f6ccf8518914e9a%7C0%7C0%7C638570397978366391%7CUnknown%7CTWFpbGZsb3d8eyJWIjoiMC4wLjAwMDAiLCJQIjoiV2luMzIiLCJBTiI6Ik1haWwiLCJXVCI6Mn0%3D%7C0%7C%7C%7C&sdata=4sEfZUCxi2w2it%2BbP7Vx0gcsCJSno7DzrwuhLDY%2BXmc%3D&reserved=0).

We look forward to receiving your revised manuscript.

Kind regards,

Robert Jeenchen Chen, MD, MPH
Academic Editor
PLOS ONE


**[Note: HTML markup is below. Please do not edit.]

Reviewers' comments:

Reviewer's Responses to Questions**

**Comments to the Author

1. If the authors have adequately addressed your comments raised in a previous round of review and you feel that this manuscript is now acceptable for publication, you may indicate that here to bypass the “Comments to the Author” section, enter your conflict of interest statement in the “Confidential to Editor” section, and submit your "Accept" recommendation.**

**Reviewer #2: All comments have been addressed**

**Reviewer #4: (No Response)**

**2. Is the manuscript technically sound, and do the data support the conclusions?

The manuscript must describe a technically sound piece of scientific research with data that supports the conclusions. Experiments must have been conducted rigorously, with appropriate controls, replication, and sample sizes. The conclusions must be drawn appropriately based on the data presented.**

**Reviewer #2: Yes**

**Reviewer #4: Partly**

**3. Has the statistical analysis been performed appropriately and rigorously?**

**Reviewer #2: N/A**

**Reviewer #4: Yes**

**4. Have the authors made all data underlying the findings in their manuscript fully available?

The** [**PLOS Data policy**](https://can01.safelinks.protection.outlook.com/?url=http%3A%2F%2Fwww.plosone.org%2Fstatic%2Fpolicies.action%23sharing&data=05%7C02%7Cmelany.gaetani%40sickkids.ca%7C738234050cea4e39418308dca864c63b%7C3961553ff47e49eb9f6ccf8518914e9a%7C0%7C0%7C638570397978375268%7CUnknown%7CTWFpbGZsb3d8eyJWIjoiMC4wLjAwMDAiLCJQIjoiV2luMzIiLCJBTiI6Ik1haWwiLCJXVCI6Mn0%3D%7C0%7C%7C%7C&sdata=e4XWDRhheCnJbbv%2Fl5xJ6UONZBHevI8cl8nLezclENc%3D&reserved=0) **requires authors to make all data underlying the findings described in their manuscript fully available without restriction, with rare exception (please refer to the Data Availability Statement in the manuscript PDF file). The data should be provided as part of the manuscript or its supporting information, or deposited to a public repository. For example, in addition to summary statistics, the data points behind means, medians and variance measures should be available. If there are restrictions on publicly sharing data—e.g. participant privacy or use of data from a third party—those must be specified.**

**Reviewer #2: Yes**

**Reviewer #4: Yes**

**5. Is the manuscript presented in an intelligible fashion and written in standard English?

PLOS ONE does not copyedit accepted manuscripts, so the language in submitted articles must be clear, correct, and unambiguous. Any typographical or grammatical errors should be corrected at revision, so please note any specific errors here.**

**Reviewer #2: Yes**

**Reviewer #4: No**

**6. Review Comments to the Author

Please use the space provided to explain your answers to the questions above. You may also include additional comments for the author, including concerns about dual publication, research ethics, or publication ethics. (Please upload your review as an attachment if it exceeds 20,000 characters)**

**Reviewer #2: suitable for publication. All comments are appropriate and all questions addressed. No other concern raised**

Thank you. We appreciate the Reviewer’s comments and revisions.

**Reviewer #4: PONE-D-23-38349R2
Vascular Access Devices and Associated Complications in Paediatric Critical Care: A Prospective Cohort Study

Thank you for your submitted manuscript for peer review at PLOS ONE.

This is STROBE-compliant study of pediatric patients who had a vascular access device inserted during a 4.5 yr period in a PICU, with a focus on complications specific to device insertion, lumen occlusion, leaking (which is quite non-specific in its overall definition) and other associated outcomes.

I was not an initial reviewer for the prior revisions until this R2, however I have some concerns regarding the manuscript.

I have highlighted the areas that require further attention before acceptance for publication.

ABSTRACT: overall lacking, however what the conclusion is missing is its summary of the findings - this is usually the first thing I read in an abstract is the conclusion, as this would tell me what the authors outcome recommendations were based upon their study findings - however this does not.**

**Please avoid bland, generic conclusions like “a wide breadth of device and lumen complications that affect critically ill children. Future work describing strategies that resolve or limit complications are needed to address current knowledge gaps..” - we know that almost all authors make these propositions for future research.. just provide a short but detailed summary of your works findings and save that stuff for the main conclusion.**

Thank you. We have added more findings and significantly revised the conclusion of the abstract.

“Approximately 1 in 5 vascular devices had one or more complication in ICU, most devices continued to be used without removal, and some complications resolved. The implications of the fluids infused to maintain device function warrants further study, as do strategies to resolve or limit the their complications in children.

**There is no study timeline mentioned in the abstract methods section, just the facility type. This should be included for clarity. UPDATE: from main manuscript, I found May 2014-Dec 2018 (4.5yrs) - just add 4.5yrs for general study period in the abstract.**

Thank you. This has been added.

“An observational cohort study was conducted over a 4.5 years period, in two quaternary, university-affiliated paediatric intensive care units (ICU).”

**KEYWORDS: Please standardize the use of MeSH terms wherever available - details can be found at** [**https://meshb.nlm.nih.gov/**](https://meshb.nlm.nih.gov/) **MeSH helps standardize search terms to ensure greater searchability.**

Thank you. The terms are MeSH standardized.

**Please avoid the use of first-person perspective (we, our, etc.) and use third-person (this study, the authors, this research, these findings, the outcomes, etc.) as this creates a more academically and less personalized styled manuscript.**

Thank you. These terms have been replaced throughout.

**METHODOLOGY:
Seldinger technique is used for CICC’s - PICCs are inserted using MST (Modified Seldinger Technique) - please correct this oversight so that the correct insertion technique is described for these specific devices.**

Thank you. This has been changed.

“In this center, CVCs and PICCs are most commonly inserted using ultrasound guided, modified Seldinger technique, however a significant proportion of patients have pre-existing temporary or long term vascular access devices on admission.”

**I think this really should have been an IRB/ethics approved study - regardless of the ‘observational’ aspect of the study, all patients who had a CICC/PICC placed “prospectively” had an invasive procedure performed that would have required written or verbal consent/assent for the procedure - if it was a restrospective study, it would have been different. This is in alignment with any human research as per the Declaration of Helsinki.**

Prior to starting this study was reviewed from scientific and research ethics perspectives and its conduct was approved. The research ethics board approval number is within this version of the manuscript. Thank you.

“Permission to conduct the study was provided by the Hospital for Sick Children Research Ethics Board (Approval number: 1000006271).”

**RESULTS:
The number of reported devices for a PICU over 4.5 yrs appears to be relatively low in N (220). Is there a reason why?**

Thank you. The studied sample was one of convenience. This has been more explicitly described in the methods and is why we focused on descriptions within patients rather than rates of device utilization per ICU admission. A statement that enrolment occurred on the days of observation has been added to the methods. We note 220 is the number of patients observed in whom there were 683 devices and 924 lumens.

“A total of 220 patients, 683 devices and 924 lumens were observed.”

**The authors chose to firstly report device days, which can be useful depending on the situation, however it would be worthwhile to report the total number of devices in the study, broken down by each device type.**

Thank you. We agree with the reviewer that amalgamated numbers are important to report as a first step. We report the number of total devices and lumens in the first sentence of results, as the central value in figure one and in a hierarchical fashion in table one. We also agree that each device type has unique considerations and for that reason all other analysis have been broken down by device type (Figure 2, Figure 3, Supp table 3, Supp table 4).

**Reporting generic complication rates is lacklustre. It doesnt truely explain the underlying rationale for these reported complications.**

Thank you. We agree that *only* reporting complication rates is not as useful. For this reason in addition to the overall complication rates we report [1] complication type by device (Figure 2), [2] the time to complication by device and complication type (Figure 3); [3] the estimated probability of a device transitioning from no complications, to having a complication to then continued use or being removed over a given period (Supp table 3) and [4] finally, we evaluate relationships of various covariates with state change -from complication free to complication, removal or complication resolution (Supp table 4). We believe the inclusion of a multi-state model for device complications is in itself relatively novel, and that our findings that complications may resolve is also novel.

We hope this extensive, stratified and time anchored analysis adds context and useable information to clinicians at the bedside that moves beyond generic complication rates.

**Also stating that the most common PIVC complication was removal is useless. Removal is the end-use of the device. It would be more meaningful if the authors stated what was the reason for PIVC removal - accidental/patient initiated, infiltration, extravasation, infection, etc.. these are the underlying reasons for accurate PIVC removal criteria and help define the causal effects.**

Thank you. As the reviewer points out the rationale for PIV removal would be an interesting component of the PIV information. In our centre, PIVs are seldom removed if working well and therefore removal was included as a complication. This reasonable assumption has been explicitly stated as limitation of the approach used in this work.

“Fourth, we also did not describe the reason for removal or long-term complications of access devices. Our anecdotal experience suggests that removal of functional peripheral catheters is uncommon, and we prioritized available study resources accordingly. Future work explicitly describing reasons for removal may help identify delayed and premature removal and associated clinical consequences.”

**There is a fair amount of discussion regarding accessory devices (stopcocks, pumps, Y-connectors, etc.) however these are not anlaysed in the tabulated results, which may provide a more impactful understanding of where these complications may be occuring.**

Thank you. We agree with the reviewer that this would be interesting to analyze however for stratified analyses (important as outlined above) there were too few specific device-types to permit the conduct of meaningful comparisons. We have included this as a limitation.

“Fifth, there were few tunneled devices, dialysis catheters and umbilical venous catheters limiting the robustness of estimates from these devices and the modest number of specific device types precluded meaningful analyses of specific devices and device-configurations as in previous studies.”

**4:2:1 rule - what is this? Where are the references that cite this is an empirical protocol that is evidence based? Please provide references to support your statements. This also applies throughout the entire paper as there are other areas that need reference support.**

Thank you. We have now referenced all places where the 4:2:1 rule is referred to in the manuscript. This is a paediatric calculation is frequently used for the volume of maintenance intravenous fluids prescribed to children. We have provided a reference for readers wishing to further explore this practice.

**The reference of heparinized saline for PIVCs cites neonatal research, which is a different patient population to pediatrics/children.
I would consider using more age-specific/pediatric research to support your statements.
e.g.
Kleidon TM, Schults J, Rickard CM, Ullman AJ. Techniques and technologies to improve peripheral intravenous catheter outcomes in pediatric patients: systematic review and Meta‐Analysis. Journal of Hospital Medicine. 2021 Dec;16(12):742-50.

Zhang W, Wei B, Chai M, Chen D. Heparin versus normal saline for the care of peripheral intravenous catheters in children: A meta‐analysis. Nursing Open. 2024 Jan;11(1):e2045.

Dobrescu A, Constantin AM, Pinte L, Chapman A, Ratajczak P, Klerings I, Emprechtinger R, Allegranzi B, Zingg W, Grayson ML, Toledo J. Effectiveness and safety of measures to prevent infections and other complications associated with peripheral intravenous catheters: A systematic review and meta-analysis. Clinical Infectious Diseases. 2024 Jun 15;78(6):1640-55.

Indarwati F, Munday J, Keogh S. Peripheral intravenous catheter insertion, maintenance and outcomes in Indonesian paediatric hospital settings: A point prevalence study. Journal of Pediatric Nursing. 2023 Nov 1;73:106-12.

Pacilli M, Bradshaw CJ, Clarke SA. Use of 8-cm 22G-long peripheral cannulas in pediatric patients. The Journal of Vascular Access. 2018 Sep;19(5):496-500.**

We thank the reviewer for their supportive response and suggested references. We have incorporated some of the more age-specific/pediatric research. For example referencing Pacilli et al as it refers to a type of peripheral access that was not routinely used in our unit during the study period and added reference to an RCT of 12 vs 24 hourly flushing of peripheral venous catheters in hospitalised children (Schreiber, ADC 2014). We have removed a number of the earlier references.

**Please ensure JMIR reporting of P-values in both the abstract and main manuscript, including all tables and figures.

P value is always italicized and capitalized with no spaces between characters.
Do not use 0 before the decimal point for statistical values P, alpha, and beta because they cannot equal 1, in other words, write P<.001 instead of P<0.001**

**The actual P value* should be expressed (P=.04) rather than expressing a statement of inequality (P<.05), unless P<.001.**

**P values should not be listed as not significant (NS) since, for meta-analysis, the actual values are important and not providing exact P values is a form of incomplete reporting.**

**If P>.01 then the P value should always be expressed to 2 digits whether or not it is significant. When rounding, 3 digits is acceptable if rounding would change the significance of a value (eg, you may write P=.049 instead of .05).**

**If P<.01, it should be expressed to 3 digits.
For P values less than .001, report them as P<.001, instead of the actual exact P value. Expressing P to more than 3 significant digits does not add useful information since precise P values with extreme results are sensitive to biases or departures from the statistical model.
P=.000 (as outputted by some statistical packages) is impossible and should be written as P<.001
This should be in the main body of text as well as all figures/tables where P-values are stated.**

Thank you. The formatting has been updated throughout to comply with the above guidelines.

**Please replace the term “line/lines” throughout the manuscript/tables/figures with the correct terminology/nomenclature ‘catheters’ or ‘devices’ - “line/lines” is an unfortunate frequently used non-descriptive term that is ambiguous in describing venous catheters. Correct nomenclature and terminology are essential in the proper description of medical devices and yet we continue to use/see the term ‘line/lines' to describe various invasive devices; central venous catheters ("central lines”), peripherally inserted central catheters ("PICC lines”), femoral catheters ("femoral lines”), arterial catheters (arterial lines).. a line is nothing but that - a line!**

Thank you. This has been updated throughout.

**REFERENCES;
20/30 (66%) > 5yrs old with 10/30 (33%) >10yrs old.
I woud caution on the frequent citing/use of older literature, especially from the pre-ultrasound era, as procedural complications have been positively impacted with the upsurge in US use for procedureal safety and procedural efficiencies. I have provided a number of publications that would provide additional support to your submission.

Some further suggested readings;
Nickel B, Gorski L, Kleidon T, Kyes A, DeVries M, Keogh S, Meyer B, Sarver MJ, Crickman R, Ong J, Clare S. Infusion therapy standards of practice. Journal of Infusion Nursing. 2024 Jan 1;47(1S):S1-285. DOI: 10.1097/NAN.0000000000000532

Pittiruti M. Vascular access in pediatric patients: classification and indications. InVascular access in neonates and children 2022 Jun 4 (pp. 3-24). Cham: Springer International Publishing.

Barone G, D’Andrea V, Ancora G, Cresi F, Maggio L, Capasso A, Mastroianni R, Pozzi N, Rodriguez-Perez C, Romitti MG, Tota F. The neonatal DAV-expert algorithm: a GAVeCeLT/GAVePed consensus for the choice of the most appropriate venous access in newborns. European Journal of Pediatrics. 2023 Aug;182(8):3385-95.

Spencer TR, Pittiruti M. Rapid Central Vein Assessment (RaCeVA): a systematic, standardized approach for ultrasound assessment before central venous catheterization. The journal of vascular access. 2019 May;20(3):239-49.

Pittiruti M, Annetta MG, D’andrea V. Point-of-care ultrasound for vascular access in neonates and children. European Journal of Pediatrics. 2024 Mar;183(3):1073-8.

Crocoli A, Martucci C, Persano G, De Pasquale MD, Serra A, Accinni A, Aloi IP, Bertocchini A, Frediani S, Madafferi S, Pardi V. Vascular access in pediatric oncology and hematology: state of the art. Children. 2022 Jan 5;9(1):70.

Georgeades C, Rothstein AE, Plunk MR, Van Arendonk K. Iatrogenic vascular trauma and complications of vascular access in children. InSeminars in Pediatric Surgery 2021 Dec 1 (Vol. 30, No. 6, p. 151122). WB Saunders.

Zito Marinosci G, Biasucci DG, Barone G, D’Andrea V, Elisei D, Iacobone E, La Greca A, Pittiruti M. ECHOTIP-Ped: a structured protocol for ultrasound-based tip navigation and tip location during placement of central venous access devices in pediatric patients. The Journal of Vascular Access. 2023 Jan;24(1):5-13.

Pittiruti M, Celentano D, Barone G, D’Andrea V, Annetta MG, Conti G. A GAVeCeLT bundle for central venous catheterization in neonates and children: a prospective clinical study on 729 cases. The Journal of Vascular Access. 2023 Nov;24(6):1477-88.

Pittiruti M, Crocoli A, Zanaboni C, Annetta MG, Bevilacqua M, Biasucci DG, Celentano D, Cesaro S, Chiaretti A, Disma N, Mancino A. The pediatric DAV-expert algorithm: A GAVeCeLT/GAVePed consensus for the choice of the most appropriate venous access device in children. The Journal of Vascular Access. 2024:11297298241256999.

Annetta MG, Celentano D, Zumstein L, Attinà G, Ruggiero A, Conti G, Pittiruti M. Catheter-related complications in onco-hematologic children: A retrospective clinical study on 227 central venous access devices. The Journal of Vascular Access. 2024 Mar;25(2):512-8.

Paterson RS, Chopra V, Brown E, Kleidon TM, Cooke M, Rickard CM, Bernstein SJ, Ullman AJ. Selection and insertion of vascular access devices in pediatrics: a systematic review. Pediatrics. 2020 Jun 1;145(Supplement_3):S243-68.

Ullman AJ, Gibson V, Takashima MD, Kleidon TM, Schults J, Saiyed M, Cattanach P, Paterson R, Cooke M, Rickard CM, Byrnes J. Pediatric central venous access devices: practice, performance, and costs. Pediatric Research. 2022 Nov;92(5):1381-90.

Kleidon TM, Doellman D, Pitts S, Stranz M. Vascular access by specialists. Pediatrics. 2020 Jun 1;145(Supplement_3):S285-7.**

We thank the Reviewer for the reference recommendations. We have reviewed these and updated the references to be more contemporary.

**SUPPLEMENTARY DOC:
S1 Table: Outcome definitions - no references to define where these definitions came from. Please address and cite appropriate resources/references.

A number of definitions can be found in the 2024 INS Standards of Practice - Nickel B, Gorski L, Kleidon T, Kyes A, DeVries M, Keogh S, Meyer B, Sarver MJ, Crickman R, Ong J, Clare S. Infusion therapy standards of practice. Journal of Infusion Nursing. 2024 Jan 1;47(1S):S1-285. DOI: 10.1097/NAN.0000000000000532**

Thank you. These were defined a priori by expert opinion to help better delineate and understand the clinical impacts of device versus lumen occlusion. Reassuringly, all complications outlined in the above reference under section 7: Device complications are included in the definitions. We have added this to the methods for clarity.

“The main outcomes were defined a priori and describe type and timing of device complications and lumen malfunctions.”

**OVERALL IMPRESSION: I think the authors have a significant amount of revision that is still due with this submission to get it to an acceptable level for publication. I have provided a number of comments regarding specific issues that need to be clearly addressed. While there is no doubt of the importance of the topic, especially in children, this research has been written with amore wideangle lens than drilling down into the cuasal effects of specific complications. I think this would be a more useful process by performing a deep-dive evaluation of the different devices included in the study, rather than just skimming the surface.**

We thank the Reviewer for their time and thoughtful responses. We hope that the extensive analysis undertaken to include a stratified analysis by device type, detailing not only the rates of complications but also the types of complications associated with each device is helpful. Additionally, we provided a comprehensive description of survival of devices and estimated probabilities for different states (removal, use, no complications) specific to each device type which is immediately useful information to integrate at the bedside. We believe these aspects address the need for a more in-depth examination and appreciate your input in refining our approach for our ongoing work.

**7. PLOS authors have the option to publish the peer review history of their article (**[**what does this mean?**](https://can01.safelinks.protection.outlook.com/?url=https%3A%2F%2Fjournals.plos.org%2Fplosone%2Fs%2Feditorial-and-peer-review-process%23loc-peer-review-history&data=05%7C02%7Cmelany.gaetani%40sickkids.ca%7C738234050cea4e39418308dca864c63b%7C3961553ff47e49eb9f6ccf8518914e9a%7C0%7C0%7C638570397978382074%7CUnknown%7CTWFpbGZsb3d8eyJWIjoiMC4wLjAwMDAiLCJQIjoiV2luMzIiLCJBTiI6Ik1haWwiLCJXVCI6Mn0%3D%7C0%7C%7C%7C&sdata=b8RV8vRAIxPa6AYGvGUNSVLh5BemCI24XXTB5fyvxgw%3D&reserved=0)**). If published, this will include your full peer review and any attached files.

If you choose “no”, your identity will remain anonymous but your review may still be made public.

Do you want your identity to be public for this peer review? For information about this choice, including consent withdrawal, please see our** [**Privacy Policy**](https://can01.safelinks.protection.outlook.com/?url=https%3A%2F%2Fwww.plos.org%2Fprivacy-policy&data=05%7C02%7Cmelany.gaetani%40sickkids.ca%7C738234050cea4e39418308dca864c63b%7C3961553ff47e49eb9f6ccf8518914e9a%7C0%7C0%7C638570397978387899%7CUnknown%7CTWFpbGZsb3d8eyJWIjoiMC4wLjAwMDAiLCJQIjoiV2luMzIiLCJBTiI6Ik1haWwiLCJXVCI6Mn0%3D%7C0%7C%7C%7C&sdata=1VKV2Qj0UckxP0wX4CexNxad%2FQD6IqbmFE2ou8PHzxw%3D&reserved=0)**.**

**Reviewer #2: No**

**Reviewer #4: No**

The authors thank the reviewers and the editor for sharing their thoughts and suggestions and for the opportunity to respond. We believe the manuscript has continued to improve. We appreciate the newer reviewers input.

Respectfully,

C. Parshuram for the authors
